# Treatment of ErbB2 breast cancer by mitochondrial targeting

**DOI:** 10.1186/s40170-020-00223-8

**Published:** 2020-07-14

**Authors:** Sophia Eldad, Rachel Hertz, Gilad Vainer, Ann Saada, Jacob Bar-Tana

**Affiliations:** 1 Dept of Human Nutrition and Metabolism, Hebrew University Medical School, 91120 Jerusalem, Israel; 2 Dept of Pathology, Hadassah-Hebrew University Medical Center, 91120 Jerusalem, Israel; 3 Department of Genetics and Metabolic Diseases, Hadassah-Hebrew University Medical Center, 91120 Jerusalem, Israel

**Keywords:** Breast cancer, ErbB2, mTORC1, Lipid rafts, Mitochondria

## Abstract

**Background:**

ErbB2 breast cancer still remains an unmet need due to primary and/or acquired resistance to current treatment strategies. MEDICA compounds consist of synthetic long-chain α,ω-dicarboxylic acids previously reported to suppress breast cancer in PyMT transgenic mice.

**Methods:**

MEDICA efficacy and mode of action in the ErbB2 context was studied in ErbB2 transgenic mice and human breast cancer cells.

**Results:**

MEDICA treatment is shown here to suppress ErbB2 breast tumors and lung metastasis in ErbB2/neu MMTV transgenic mice, to suppress ErbB2/neu xenografts in nod/scid mice, and to suppress survival of AU565 and BT474 human ErbB2 breast cancer cells. Suppression of ErbB2 breast tumors by MEDICA is due to lipid raft disruption with loss of ErbB family members, including EGFR, ErbB2, and ErbB3. In addition, MEDICA inhibits mTORC1 activity, independently of abrogating the ErbB receptors and their signaling cascades. The double hit of MEDICA in abrogating ErbB and mTORC1 is partly accounted for by targeting mitochondria complex I.

**Conclusions:**

Mitochondrial targeting by MEDICA suppresses ErbB2 breast tumors and metastasis due to lipid raft disruption and inhibition of mTORC1 activity. Inhibition of mTORC1 activity by MEDICA avoids the resistance acquired by canonical mTORC1 inhibitors like rapalogs or mTOR kinase inhibitors.

## Background

Breast cancer is the most common female cancer and the second leading cause of cancer-related death in women in developed countries [1]. ErbB2 (HER2) breast tumors comprise 20–25% of breast cancer cases, half of which are also hormone-receptor positive (Luminal B). Amplification of ErbB2 leads to constitutive ligand-independent ErbB2 homodimerization or heterodimerization with ErbB family members (ErbB1/EGFR, ErbB3), leading to recruitment and activation of the PI3K/Akt/mTORC1, MEK/ERK/mTORC1, and c-Src/JAK/STAT3 transducers that control cell proliferation, survival, angiogenesis, and metastasis. ErbB2 breast cancer is treated with chemotherapy, surgery, and ErbB2-targeting agents (e.g., trastuzumab, prastuzumab, lapatinib) [1–3]. However, ~ 20% of ErbB2 patients present primary resistance to ErbB2 targeting agents and ~ 70% of patients with ErbB2 metastatic breast cancer who initially respond acquire resistance within 1 year [2]. Hence, in spite of the advances accomplished in treating ErbB2 breast cancer, the ErbB2 challenge still remains an unmet need.

Breast cancer is mostly diagnosed in postmenopausal women aged 50 and above, namely, a population heavily inflicted by type 2 diabetes (T2D) or prediabetes (50% prevalence in females aged ≥ 50 [4]). Indeed, T2D increases the relative risk of breast cancer morbidity and mortality by 1.2- and 1.8-fold [5], respectively. The increase in breast cancer due to T2D is ascribed to basal insulin resistance/hyperinsulinemia, with insulin acting as mitogenic growth factor. In line with that, insulin-resistant hyperinsulinemic animal models inoculated with mouse mammary carcinoma cells present advanced mammary tumors [6, 7]. Similarly, treatment of T2D patients with insulin or insulin secretagogues (e.g., sulfonylurea) is associated with significantly higher risk of breast cancer [8, 9], whereas retrospective clinical studies have indicated improved disease-free and overall survival of diabetic ErbB2 breast cancer patients treated with insulin sensitizers (metformin, thiazolidinediones) [10–13]. Increase in survival due to metformin and metformin-analogues has also been reported in transgenic ErbB2/neu mice [14–16], implying that add-on insulin sensitizers may improve survival of non-diabetic ErbB2 breast cancer.

MEDICA compounds consist of synthetic long-chain α,ω-dicarboxylic acids, substituted in the αα′ or ββ′ carbons [HOOC-C(α′)-C(β′)-(CH_2_)_n_-C(β)-C(α)-COOH, *n* ≥ 10] [17–21]. MEDICA analogues may be thio-esterified endogenously to their respective CoA-thioesters, but in contrast to natural long-chain fatty acids (LCFA), these compounds are not incorporated into lipids, while the substitutions at the αα′ or ββ′ positions block their β-oxidation. MEDICA analogues are mostly excreted in bile as respective glucuronides. MEDICA compounds simulate LCFA in activating AMP-activated protein kinase (AMPK) (being 20-folds more potent than metformin) [17] and in suppressing adenylate cyclase [19]. MEDICA compounds proved potent anti-diabetic efficacy in type II and I diabetic animal models [17, 18, 20], while suppressing diabetes-induced colorectal cancer [21]. Also, MEDICA treatment has previously been reported by us to suppress triple-negative breast tumor growth and lung metastasis of mice and cells expressing the polyoma middle T antigen (PyMT) driven by the mammary MMTV promoter (MMTV-PyMT) [22]. These considerations prompted our interest to study MEDICA activity in the ErbB2 breast cancer context. MEDICA treatment is shown here to suppress ErbB2 breast cancer in vivo and cell lines by targeting mitochondrial oxidative phosphorylation, resulting in suppression of ErbB family members and inhibition of mTORC1 activity.

## Methods

### Animals and diets

FVB-tg(MMTV-ErbB2) female mice (Jackson Laboratory) express activated rat ErbB2 (*c-neu,* V664G) oncogene under the direction of the mouse mammary tumor virus (MMTV) promoter [23]. Mice were kept in standard SPF conditions in 12-h light/dark periods, with free access to food and water. Four-month-old mice were fed for 8 weeks with either regular chow or MEDICA in feed (0.04%W/W). Upon sacrifice, mice were anesthetized using ketamine/xylazine; breasts were photographed, dissected, and weighed; and breast tumors and lungs were immediately frozen in liquid nitrogen for RNA and protein analysis. Tumor volume was estimated by measurement of width and length of breast tumor foci and calculated by the formula 4π/3(((*W* + *L*)/4)^3^). Lung metastasis was determined by rat ErbB2 neu transcript by qRT-PCR.

Immortalized ErbB2/neu cells from FVB MMTV ErbB2 neu breast tumors (RH2111 cells) were subcutaneously xenografted to NOD/SCID (Jackson Laboratory) female mice (5 × 10^6^ cells per mouse). Tumors were visible 1 week after injection. Mice with visible tumors were fed with either regular chow or MEDICA in feed (0.04%W/W), followed by caliper monitoring tumor volumes for 30 days. Mice were anesthetized using ketamine/xylazine, and tumors were sampled and weighed.

### Cultured cells

Human AU565 (ATCC CRL-2351) and BT474 (ATCC HTB-20) cells were cultured in RPMI-1640 (Biological Industries, Beit Haemek, Israel) supplemented with 10% fetal calf serum, 2 mM l-glutamine, and penicillin/streptomycin solution at 37 °C in humidified atmosphere containing 5% CO2 in the presence of additions as indicated. Human MCF10 cells were cultured in DMEM/F12 (Biological Industries, Beit Haemek, Israel) supplemented with 5% horse serum, 20 ng/ml EGF, 0.5 μg/ml hydrocortisone, 100 ng/ml cholera toxin, 10 μg/ml insulin, and penicillin/streptomycin solution at 37 °C in humidified atmosphere containing 5% CO2. RH2111 cells were derived from FVB MMTV ErbB2 neu breast tumors. Excised tumors were disintegrated in serum-free mammary epithelial cell growth medium (MEGM^TM^) (Lonza). Fibroblasts were removed by 3–4 rounds of trypsinization, yielding cells that displayed exclusive epithelial morphology over subsequent passages. RH2111 cells were cultured in DMEM/F12 medium supplemented with 10% fetal calf serum in the presence of additions as indicated. Trastuzumab-resistant AU565 and BT474 cells were derived by continuously culturing parental cells for 4 months with increasing concentrations of trastuzumab (Roche) up to 5 μg/ml. Resistant cells were maintained in 4 μg/ml trastuzumab. Cells were routinely tested for Mycoplasma by the EZ-PCR kit (Biological Industries, Beit Haemek, Israel). Cell growth was quantified using the methylene blue assay. Where indicated, cells were cultured in medium containing 10 mM galactose instead of glucose. Where indicated, EGF (50 ng/ml) was added during the last 15 min of incubation.

### Anchorage-dependent colony assay

Cells seeded on 60-mm plates (5000cells/well) were allowed to form colonies for 18 days in the presence of added MEDICA as indicated. Colonies were fixated with 0.625% glutaraldehyde and stained with methylene blue. Colonies that reached ≥ 400um in size were counted.

### Soft agar colony assay

Cells were seeded on 0.5% bottom agar 60-mm plates with culture medium containing 0.3% agar and 5000 cells. Cells were incubated with 0, 150 μM, and 200 μM MEDICA for 25 days. Colonies that reached ≥ 100um in size were counted.

### Tumor spheroids

BT474 spheroids were generated in polyHEMA-coated U-shaped 96-well plates. Fifty microliters of 0.5% polyHEMA (Sigma) in 95% ethanol was added to each well and allowed to evaporate in 37 °C. 4–5 × 10^3^ BT474 cells, or BT474 cells infected with empty or NDI1 virus as indicated, were added to each well in 50 μl RPMI-1640 medium supplemented with 10% fetal calf serum, 2 mM l-glutamine, penicillin/streptomycin solution, and 2% matrigel, and the plate centrifuged at 250*g* for 5 min. Spheroids were allowed to form and were treated as indicated. Spheroid viability was assayed by acid phosphatase [23].

### Lenti- and retrovirus infections

Human AMPKα1/AMPKα2 ShRNA was from Jones RG (Goodman Cancer Research center, McGill University Montreal Canada). ShREDD1 (NM-019058) was from Sigma Mission. ShSestrin2 was from Budanov AV (Department of human and molecular genetics Virginia Commonwealth University VA, USA). NDI1 plasmid was from Addgene. Cells infected with control virus or shAMPK, shSestrin2, or shREDD1 plasmids were selected by puromycin. Cells infected with empty or NDI1 were selected by blasticidine.

### Cell cycle distribution

Cells were trypsinized, washed with cold PBS, suspended in PBS/70% ethanol, and kept at − 20 °C. For FACS analysis, cells were centrifuged, washed with PBS, and suspended in 700 μl propidium iodide (PI)/Triton X-100/RNAase A staining solution (20 μg/ml PI, 0.1% Triton X-100, 0.1 mg/ml RNAase A in PBS). Cell cycle distribution was analyzed using FACScan (BD Biosciences).

### Immunofluorescence

Cells were grown on cover slips and treated as indicated. Following treatment, cells were rinsed with PBS and fixed with 4% paraformaldehyde for 30 min, permeabilized with 0.1% Triton X-100 in 1% FBS for 5 min, and blocked with 0.1% FBS for 30 min. Fixated cells were incubated overnight with primary antibodies for EGFR (1:50), ErbB2 (1:100), or caveolin 1 (1:100) at 4 °C, followed by incubation with the secondary antibody Cy-3 conjugated donkey anti rabbit IgG (1:300) (Jackson Immunoresearch). Slides were mounted with DAPI 2ug/ml for nuclei visualization. Fluorescent intensity was analyzed by confocal microscopy (Zeiss LSM 710; Axioobserver Z1).

### Biotin tagging of plasma membrane proteins

Cells were treated as indicated and rinsed on ice three times with PBSCM (PBSx1 pH 8.0, 0.5 mM CaCl, 1 mM MgCl_2_), followed by adding non-permeable Sulfo-NHS-SS-Biotin (Thermo Scientific) in PBSCM (0.5 mg/ml) for 15 min. Cells were then rinsed with PBSCM, quenched for 10 min at 4 °C with glycine buffer (PBSx1 pH 8.0, 0.1 M Tris pH 8.0, 192 mM glycine), and rinsed twice with PBSx1 pH 8.0, followed by lysing the cells in lysis buffer [24]. Cells were then centrifuged at 12,000*g* for 15 min at 4 °C, supernatant aliquot was kept at − 70 °C for further Western analysis, and the rest incubated overnight at 4 °C with 25 μl of streptavidin beads (Streptavidin Agarose Resin, Thermo Scientific). Beads were rinsed three times with lysis buffer and subjected to Western analysis [24].

### Ganglioside (GM1) staining

Cells seeded on cover slip were rinsed on ice with RPMI1640, stained on ice for 1 h in the dark with cholera toxin subunit B labeled with Alexa Fluor 555 (5 μg/ml RPMI1640), rinsed 3 times with PBS, and fixed with 4% PFA for 10 min on ice, followed by 10 min at room temperature. Slides were mounted with DAPI 2 μg/ml for nuclei visualization. Fluorescence intensity was analyzed by confocal microscopy (Zeiss LSM 710; Axioobserver Z1).

### Caveolin staining

Cells were trypsinized, centrifuged for 5 min at 1000*g*, rinsed in PBS/1 mM MgCl2 (PBSM), and centrifuged and fixated with 4% paraformaldehyde for 10 min at 4 °C, followed by 15 min at room temperature. Fixated cells were rinsed with PBSM, suspended in PBSM/3% FCS for 15 min, and incubated with anti-caveolin1 antibody for 30 min at room temperature. Cells were then rinsed in PBSM/3% FCS, suspended for 30 min in PBSM/3% FCS containing secondary antibody Cy-3 conjugated donkey anti rabbit IgG (1:200) (Jackson Immunoresearch), rinsed in PBSM/3 % FCS, and re-suspended in PBSM/3% FCS. Caveolin was analyzed using FACScan (BD Biosciences).

### GM1-rich membrane fractions

Cells were washed with ice-cold PBS, scraped with 500 mM sodium carbonate pH 11, homogenized using a loose fitting Dounce homogenizer (10 strokes), and then sonicated (three 10-s bursts; SONICS vibra cell ultrasonic processor). The homogenate was adjusted to 45% sucrose by adding 90% sucrose prepared in MBS (25 mM Mes, pH 6.5, 0.15 M NaCl), was placed at the bottom of an ultracentrifuge tube, followed by layering a 35–5% discontinuous sucrose gradient prepared in MBS containing 250 mM sodium carbonate, and centrifuged at 32,000 rpms for 21 h in an SW41 rotor (Optima L-90 K Ultracentrifuge Bekman Coulter). Gradient fractions were dot blotted on nitrocellulose and analyzed for GM1 using HRP-conjugated cholera toxin followed by ECL. For ErbB2, gradient fractions were subjected to Western blot analysis using anti-ErbB2 antibody.

### Oxygen consumption

Cells were seeded on XF 24-well microplates (Seahorse Bioscience) at 40 × 10^3^ cells/well in culture medium. Following 16 h, medium was replaced with Leibovitz L15 medium containing 2 mM glutamine and 10% fetal calf serum (FCS). One hour prior to assay, the medium was replaced by Leibovitz L15 containing 2 mM glutamine without FCS. Measurement of basal respiration in the presence of added vehicle or MEDICA was followed by sequential additions of oligomycin (2 μM), FCCP (1 μM), and antimycin A (4 μM).

### Isolation of mitochondria

Mouse brain mitochondria were isolated by differential centrifugation. Tissue was homogenized in buffer A (320 mM sucrose, 5 mM Tris-HCl, 2 mM EGTA, pH 7.4) using a Dounce homogenizer (Teflon glass) and centrifuged for 3 min at 2000*g* to remove nuclei and cell debris. The supernatant was centrifuged for 10 min at 12,000*g* at 4 °C, and the pellet was re-suspended in buffer A containing 0.02% digitonin (Sigma-Aldrich) and re-centrifuged. The mitochondrial pellet was washed again twice with buffer A and kept at − 80 °C until use.

### Activity of mitochondrial electron transport components

Enzymatic activities of respiratory chain complexes were measured at 37 °C by standard spectrophotometry [25]. Briefly, complex I was measured by rotenone-sensitive NADH-CoQ1 reduction. Complex II was measured by succinate-CoQ1 reduction. Complexes I + III were measured by NADH-cytC reduction. Complexes II + III were measured by succinate-cytC reduction. Complex IV was measured by the oxidation of reduced cytC. NADH dehydrogenase activity was measured by NADH-ferricyanide reduction. Succinic dehydrogenase (SDH) activity was measured by succinate-mediated reduction of phenazine methosulphate coupled to dichlorophenolindophenol. Citrate synthase (CS), a ubiquitous mitochondrial matrix enzyme serving as normalizer, was measured in the presence of acetyl-CoA and oxaloacetate by the release of CoASH coupled to 5′,5′-dithiobis (2-nitrobenzoic acid). Activities of mitochondrial respiratory chain complexes in the presence of added MEDICA are presented relative to respective activities in the presence of vehicle (DMSO).

### Mitochondrial superoxide production

Cells were treated with 200uM MEDICA for 5 h followed by adding 3.3 μg/ml MitoSOX (Molecular Probes) for the last 30 min. Cells were trypsinized and analyzed using FACScan (BD Biosciences).

### Cellular ATP

Cells were seeded in 96-well plates at 10 × 10^3^ cells/well and treated as indicated. Cells were then rinsed twice with RPMI followed by 50 μl of RPMI and 25 μl of lysis solution for 5 min. Fifty-microliter aliquot was subjected to ATP measurement by the ATPlite luminescence kit (PerkinElmer).

### qRT-PCR

RNA was purified from frozen mouse tissues using the Total RNA Mini Kit (Geneaid). 0.5 mg sample was used as template for cDNA synthesis using M-MLV reverse transcriptase (Invitrogen). Real-time PCR was carried out (Rotorgene, Corbett Research) using KAPA SYBR FAST qPCR MIX (Kapa Biosystems) with the following primers (5′ to 3′): rat ErbB2 (neu) (Fw:AGGAGGACGAGTCCTTGTAGT, Rev: GCTCAGAGACCTGCTTTGGA); mouse B-act:(Fw: GGCTGTATTCCCCTCCATCG, Rev: CCAGTTGGTAACAATGCCATGT). mRNA was quantified using the ΔΔCt method.

### Cell lysis and Western blotting

Cultured cells were scraped with lysis buffer (50 mM Tris HCl pH 8.0, 1% Triton X-100, 1 mM EGTA, 1 mmol/L EDTA, 150 mM NaCl, 5 mM NaPPi, 50 mM NaF, 1 mM PMSF, 1 mM Na Vanadate, 40 nM bpVfan and protease inhibitor cocktail (Sigma)). Frozen tumor samples were sonicated (three 10-s bursts) in lysis buffer (50 mM Tris HCl pH 7.4,1% NP40, 150 mM NaCl, 10 mM NaF, 1 mM Na Vanadate, 100uM PMSF, 10 mM β glycerophosphate, 40 nM bpVfan, and protease inhibitor cocktail). Cell lysates were then centrifuged for 15 min at 12,500 rpm, and tumor samples were centrifuged for 5 min at 14,000 rpm. Protein concentration was determined by BCA (Thermo Scientific). Western blots were analyzed as previously described [24].

### Antibodies and reagents

Anti-neu/HER2 (ErbB2) antibody was from Invitrogen. Anti-ErbB2, anti-ErbB3, anti-EGFR, anti-caveolin-1, anti-phospho-ERK(Tyr204), anti-ERK, anti-phospho-Stat3(Tyr705), and anti-NDRG1 antibodies were from Santa Cruz Biotechnology INC. Anti-phospho-EGFR(Tyr1068) and anti-phospho-ErbB2(Tyr1248) antibodies were from Abcam. Anti-α-tubulin, anti-β-actin, and anti-cholera toxin B subunit antibodies were from Sigma Aldrich. Anti-4EBP1, anti-AMPKα, anti-acetyl-CoA carboxylase, anti-S6 ribosomal protein, anti-p70 S6 kinase, anti-Akt, anti-phospho-4EBP1(Thr37/46), anti-phospho-Akt(Thr308), anti-phospho-Akt(Ser473), anti-phospho-acetyl-CoA carboxylase(Ser79), anti-phospho-AMPKα(Thr172), anti-phospho-p70 S6 kinase(Thr 389), and anti-phospho-S6 Ribosomal protein(Ser240/244) antibodies were from Cell Signaling. Anti-Sestrin2 and anti-REDD1 antibodies were from Proteintech. Cholera toxin subunit B (CT-B) Conjugates were from Molecular Probes. Cy^TM^3 Conjugate was from Jackson ImmunoResarch Lab. EGF was from Perprotech. MEDICA [α,α′-tetramethyl hexadecanedioic acid, HOOC-C(CH3)2-(CH2)12-C(CH3)2-COOH] was synthesized as previously described [17, 26].

### Statistics

Statistics was performed by two-tailed repeated measure analysis of variance (GraphPad). Significance (*P* < 0.05) was analyzed by unpaired t-test with Welch correction.

## Results

**Fig. 1:**
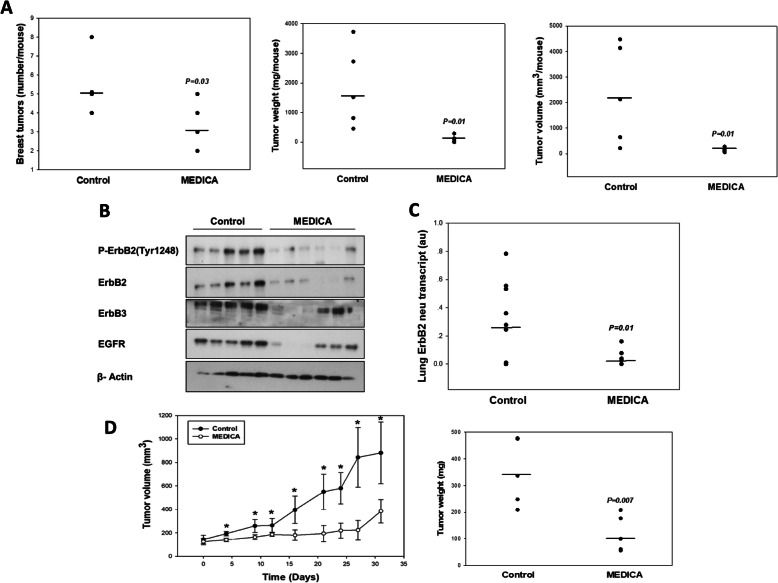
Suppression of ErbB2 breast cancer by MEDICA in vivo. **a**–**c** FVB-MMTV-ErbB2 transgenic mice were treated by MEDICA in feed as described in the “Methods” section. *Significant as compared to nontreated. **a** Breast tumor number (left), weight (middle), and volume (right). Mean (*n* = 5–6/group). **b** Tumor ErbB1, ErbB2, phospho-ErbB2(Tyr1248), and ErbB3. Representative blots. **c** Lung rat ErbB2/neu (V664G) transcript. Mean (*n* = 9–11/group). **d** NOD/SCID mice xenografted with RH2111 ErbB2 cells were treated with MEDICA in feed as described in the “Methods” section. Tumor volume (left) and tumor weight (right). Mean ± SD (*n* = 4–5/group). Significant as compared to nontreated (*P* < 0.05)

Suppression of ErbB2 breast cancer by the MEDICA analogue HOOC-C(CH_3_)_2_-CH_2_)_12_-C(CH_3_)_2_-COOH in vivo has been verified in 4-month FVB MMTV-ErbB2/neu transgenic mice [27] treated with MEDICA in feed for 8 weeks (Fig. 1). MEDICA treatment resulted in decrease in tumor number, weight, and volume (Fig. 1a); loss of ErbB1, ErbB2, and ErbB3 in breast tumors (Fig. 1b); and decrease in lung metastatic neu cells (Fig. 1c). Since breast cancer neu cells accumulate in lung blood vessels but do not produce solid lung metastases [28], metastasis was evaluated by the amount of the neu transcript in lung tissue, using rat neu-specific PCR. Suppression of ErbB2 breast cancer by MEDICA in vivo was further verified in nude mice xenografted with immortalized RH2111 ErbB2/neu cells derived from breast tumors of the FVB MMTV-ErbB2/neu transgene. MEDICA treatment resulted in decrease in RH2111 tumor volume and weight (Fig. 1d).

**Fig. 2:**
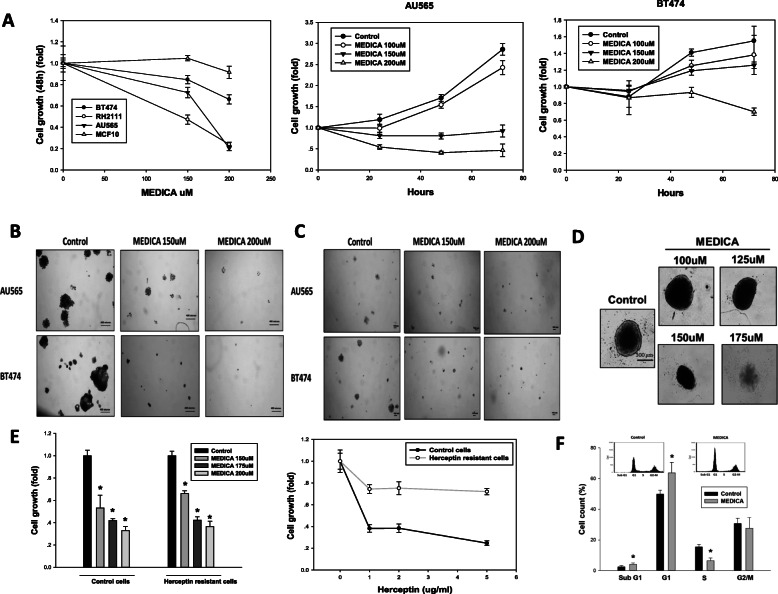
Growth inhibition of ErbB2 breast cancer cells by MEDICA. **a**–**d** Human ErbB2 AU565and BT474 cells, mouse ErbB2 RH2111 cells, and non-tumorigenic MCF10 cells were treated with MEDICA as indicated. **a** Cell growth. Mean ± SD. **b** Anchorage-dependent colonies. **c** Anchorage-independent colonies. **d** BT474 spheroids were allowed to form for 24 h followed by treatment for 8 days with MEDICA. Medium was refreshed every 3 days. Spheroid viability assayed by acid phosphatase was inhibited by 90% at 175 μM MEDICA. **e** Growth (72 h) inhibition of trastuzumab-resistant ErbB2 BT474 cells by MEDICA (left). Resistance was confirmed by growth with increasing trastuzumab concentrations as indicated (right). Mean ± SD. *Significant as compared to control (*P* < 0.05). **f** Cell cycle (24 h) suppression of AU565 cells by 200uM MEDICA. Mean ± SD. *Significant as compared to control (*P* < 0.05). Inset—representative micrographs

Suppression of ErbB2 cell growth and survival by MEDICA was further verified in mouse RH2111 ErbB2/neu cells and human breast cancer cell lines AU565 and BT474 that overexpress ErbB2. BT474 is a Luminal B cell that further expresses progesterone receptors [29]. MEDICA treatment resulted in suppressing growth of RH2111, AU565, and BT474 cells, but not the non-tumorigenic MCF10 epithelial cells (Fig. 2a). Growth suppression was further verified by formation of anchorage-dependent colonies, anchorage-independent colonies, and spheroid survival reported to be resistant to a variety of chemotherapy drugs [30] (Fig. 2b–d). Most importantly, cell growth of trastuzumab-resistant ErbB2 cells was similarly suppressed by MEDICA (Fig. 2e). Suppression of proliferation was accounted for by G1 cell cycle arrest, being accompanied by sub-G1 apoptotic cells (Fig. 2f). The μM concentrations of MEDICA reflect the high binding affinity of MEDICA to serum albumin [higher than 99%, independently of MEDICA concentrations in the range of 0–0.9 mM (Advinus N079 study)], resulting in nM concentrations of the free MEDICA acid in the culture medium.

**Fig. 3:**
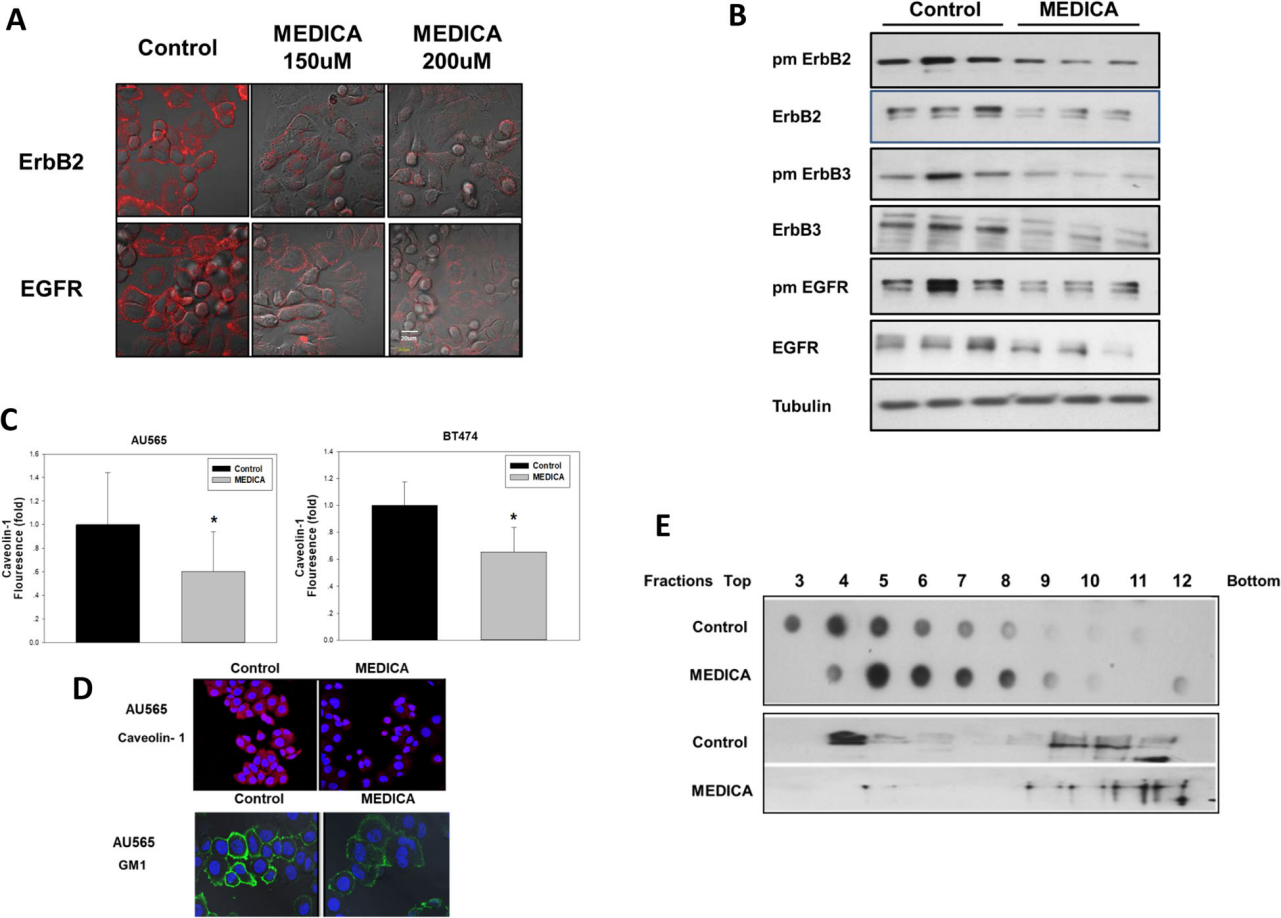
Lipid raft disruption and loss of ErbB members by MEDICA. **a** AU565 cells were treated for 24 h with MEDICA as indicated. Plasma membrane ErbB2 and EGFR were determined by immunofluorescence confocal microscopy (red). Scale bar 20 μm. **b** AU565 cells were treated for 24 h with 200uM MEDICA. Plasma membrane ErbB members were tagged with Sulfo-NHS-Biotin and isolated by Streptavidin-Agarose beads. Plasma membrane (pm) and total ErbB members were analyzed by Western blot. **c**, **d** Lipid raft disruption by MEDICA. AU565 and BT474 cells were treated for 24 h and 40 h with 200uM MEDICA, respectively. Caveolin-1 was determined by FACS and by immunofluorescence confocal microscopy. GM1 was determined by immunofluorescence confocal microscopy. *Significant as compared to control (*P* < 0.05). **e** AU565 cells were treated for 24 h with 200 μM MEDICA. Cell fractions prepared by density gradient centrifugation were subjected to GM1 (dot blot, upper frame) and ErbB2 (Western blot, lower frame) determination. Representative blots

In line with MEDICA effect in suppressing ErbB family members in transgenic ErbB2 breast cancer mice in vivo (Fig. 1b), loss of ErbB members and suppression of their respective transduction pathways were further verified in AU565 and BT474 human breast cancer cells. Plasma membrane ErbB members were determined by immunofluorescence (Fig. 3a) and biotin tagging (Fig. 3b) and found to be robustly decreased by MEDICA, together with decrease in respective total ErbB content (Fig. 3b). Since a variety of receptor tyrosine kinases (RTK), including ErbB members, are located in plasma membrane lipid rafts that enable close proximity of their respective transducing components [31, 32], the loss of plasma membrane ErbB members was further studied in terms of putative lipid raft disruption by MEDICA, as verified by caveolin-1 and GM1 ganglioside immunofluorescence. MEDICA treatment resulted in decreasing plasma membrane caveolin-1 and in decreasing, punctuating, and clustering surface GM1 (Fig. 3c, d) [33]. Lipid raft disruption by MEDICA was further verified by density gradient centrifugation. MEDICA treatment resulted in decreasing GM1 buoyancy, with concomitant loss of lipid raft ErbB2 (Fig. 3e). Hence, MEDICA effects in suppressing ErbB2 cell survival may be ascribed to loss of ErbB family members.

**Fig. 4:**
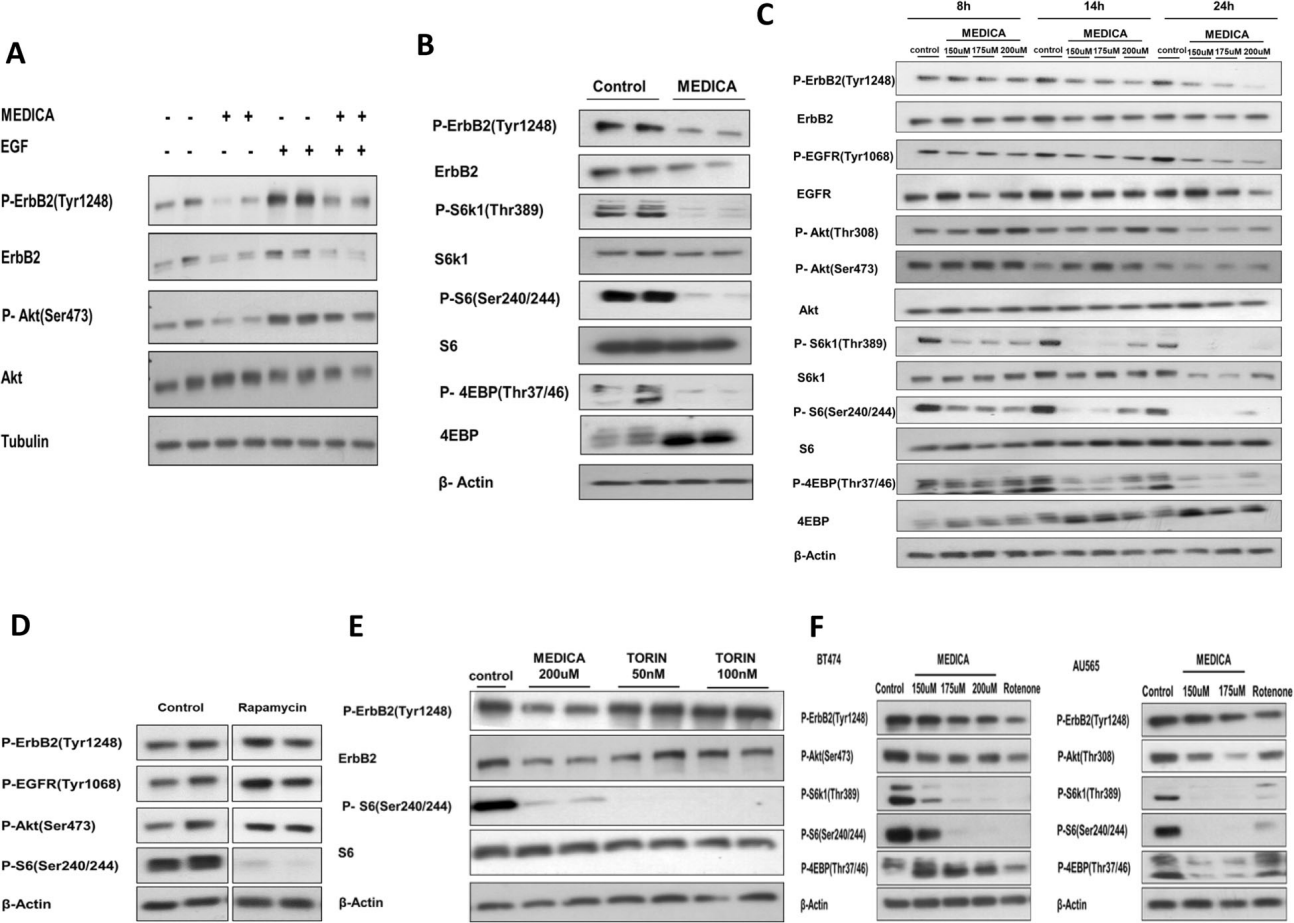
Inhibition of mTORC1 activity by MEDICA. **a** AU565 cells were treated for 24 h with 200 μM MEDICA, followed by adding 50 ng/ml EGF for the last 15 min as indicated. Representative blots. **b** AU565 cells were treated for 24 h with 200uM MEDICA. Representative blots. **c** AU565 cells were treated with MEDICA as indicated. Representative blots. **d** AU565 cells were treated for 24 h with 5 nM rapamycin. Representative blots. **e** AU565 cells were treated for 24 h with MEDICA or torin as indicated. Representative blots. **f** BT474 and AU565 cells were treated for 24 h with MEDICA and rotenone (0.5 μM) as indicated. Representative blots

Loss of ErbB members by MEDICA was accompanied by suppression of their downstream phospho-Akt(Ser473), in the basal state and upon being induced by added EGF (Fig. 4a). Inhibition of Akt by MEDICA was further accompanied by suppressing mTORC1, as verified by mTORC1 downstream substrates phospho-S6K1(Thr389), phospho-S6(Ser240/244), phospho-4EBP(Thr37/46), and total 4EBP pattern (Fig. 4b). Surprisingly however, suppression of mTORC1 activity by MEDICA could not be accounted for by loss of its ErbB drivers. Thus, inhibition of mTORC1 activity by MEDICA precedes by hours MEDICA effects in causing ErbB loss (Fig. 4c), implying that inhibition of mTORC1 activity by MEDICA is not due to loss of ErbB family members. Also, inhibition of mTORC1 activity by rapamycin (Fig. 4d) or mTOR kinase inhibitor (e.g., Torin) (Fig. 4e) does not result in loss of ErbB family members, implying that the two concerned activities of MEDICA, namely, ErbB loss and suppression of mTORC1 activity, are independently driven by an upstream cofounder.

**Fig. 5:**
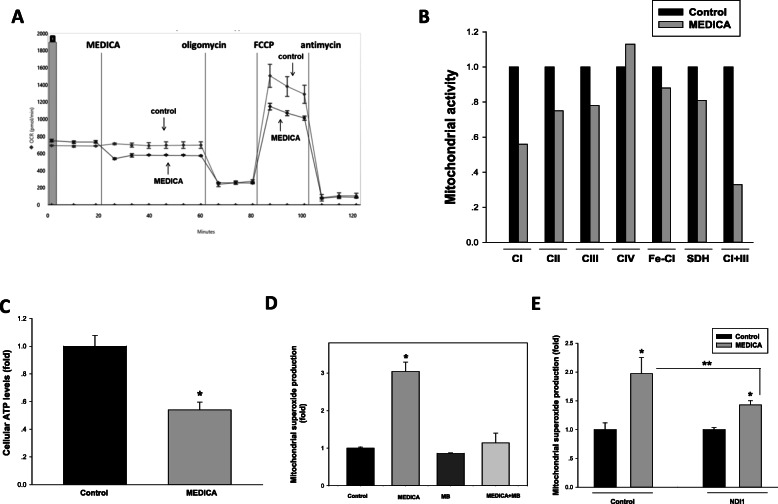
Mitochondria targeting by MEDICA. **a** Oxygen consumption rate (OCR) was determined in AU565 cells in the presence of 30 μM MEDICA as described in the “Methods” section. Representative chart. **b** Enzymatic activities of mitochondrial ETC complexes were measured in mouse brain mitochondria in the absence (denoted as 1.0) and presence of 200 μM MEDICA. CI, complex I; CII, complex II; CIII, complex III; CIV, complex IV; Fe-CI, Ferricyanide reductase; SDH, succinate dehydrogenase. Mean of 2–3 determinations. **c** ATP levels were determined in AU565 cells treated for 24 h with 200 μM MEDICA. Mean ± SD. *Significant as compared to control (*P* < 0.05). **d** Mitochondrial superoxide formation was determined in AU565 cells treated for 6 h with 200 μM MEDICA and/or 0.4 μM methylene blue (MB) as indicated. Mean ± SD. *Significant as compared to control (*P* < 0.05). **e** Mitochondrial superoxide formation was determined in BT474 cells infected with empty or NDI1 virus and treated for 6 h with 200 μM MEDICA as indicated. Mean ± SD. *Significant as compared with respective control (*P* < 0.05). **Significant as compared with empty plasmid (*P* < 0.05)

**Fig. 6:**
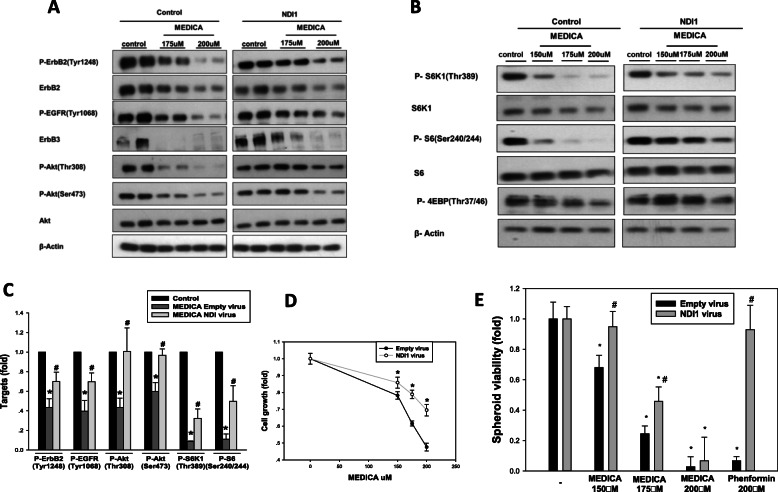
Rescue of MEDICA effects by NDI1. **a** ErbB members and Akt were measured in BT474 cells infected with empty or NDI1 virus and cultured in galactose. Cells were treated for 40 h with MEDICA as indicated. Representative blots. **b** mTORC1 downstream targets were measured in BT474 cells infected with empty or NDI1 virus. Cells were treated for 24 h with MEDICA as indicated. Representative blots. **c** Inhibition of ErbB signaling and mTORC1 by MEDICA is partly rescued by infected NDI1. Conditions as in **a** and **b**. Mean ± SD. *Significant as compared to respective control (*P* < 0.05). ^#^Significant as compared to empty virus (*P* < 0.05). **d** BT474 cells infected with empty or NDI1 virus were cultured in galactose medium and treated for 72 h with MEDICA as indicated. Mean ± SD. *Significant as compared to empty virus (*P* < 0.05). **e** BT474 spheroids prepared from cells infected with empty or NDI1 virus were allowed to form for 4 days followed by treatment for 8 days with MEDICA or phenformin as indicated. *Significant as compared to respective control (*P* < 0.05). ^#^Significant as compared to empty virus (*P* < 0.05)

Lipid raft clustering and mTORC1 activity may both be modulated by metabolic stress [34, 35]. Indeed, similarly to MEDICA, P-ErbB2(Tyr1248), P-Akt, and mTORC1 activity were suppressed by inhibition of mitochondrial complex I by rotenone (Fig. 4f), prompting our interest in studying MEDICA activity in driving mitochondrial stress and its role in ErbB loss and inhibition of mTORC1 activity. MEDICA treatment of AU565 cells resulted in inhibiting mitochondrial basal and FCCP-uncoupled oxygen consumption (Fig. 5a). Suppression of mitochondrial respiration by MEDICA was accounted for by inhibition of mitochondrial complexes I and III (Fig. 5b), accompanied by decrease in cellular ATP content (Fig. 5c) and increased mitochondrial superoxide production (Fig. 5d). MEDICA-induced mitochondrial superoxide could be partly rescued by added methylene blue that bypasses mitochondrial complexes I–III [36] (Fig. 5d) or upon by-passing mitochondrial complex I by infected yeast NADH ubiquinone reductase (NDI1) [37] (Fig. 5e). Rescue by NDI1 was further verified and better exemplified in cells cultured in galactose instead of glucose, namely growth conditions that drive metabolic dependence on mitochondrial oxidative phosphorylation [38]. Most importantly, infection with NDI1 resulted in partly rescuing MEDICA suppression of ErbB1, ErbB2, and ErbB3, and their downstream target phospho-Akt(Thr308, Ser473), as well as in rescuing MEDICA inhibition of mTORC1 downstream substrates phospho-S6K1(Thr389), phospho-S6(Ser240/244), and phospho-4EBP(Thr37/46) (Fig. 6a–c). Concomitantly, infected NDI1 resulted in partial rescue of cell growth (Fig. 6d) and spheroid viability (Fig. 6e). Hence, the effects of MEDICA in driving ErbB loss, in inhibiting mTORC1 activity, and in suppressing ErbB survival may be primarily ascribed to its mitochondrial activity. Of note, in line with MEDICA inhibition of mitochondrial complexes I and III (Fig. 5b), as contrasted with biguanides [37], infective NDI1 was fully effective in rescuing growth inhibition by phenformin, but only partly effective in rescuing growth inhibition by MEDICA (Fig. 6e).

Cell growth suppression by mitochondrial complex I inhibitors in some cancer cell lines has recently been ascribed to aspartate limitation, being rescued by added pyruvate, aspartate or by infecting aspartate transporters [39–42]. Hence, growth inhibition by MEDICA could in principle be accounted for by aspartate limitation. However, growth inhibition of AU565 or BT474 cells by MEDICA, rotenone, or phenformin was not rescued by added pyruvate or aspartate (Suppl Fig 1), implying that inhibition of AU565 or BT474 cell growth by mitochondrial complex I inhibitors is not governed by aspartate limitation. Moreover, whereas growth inhibition of A549 cells by metformin was rescued by added pyruvate or aspartate, in line with previous reports [42], inhibition of A549 cell growth by MEDICA persisted in the presence of added pyruvate or aspartate (Suppl Fig 2). Hence, growth inhibition by MEDICA surpasses aspartate limitation.

**Fig. 7:**
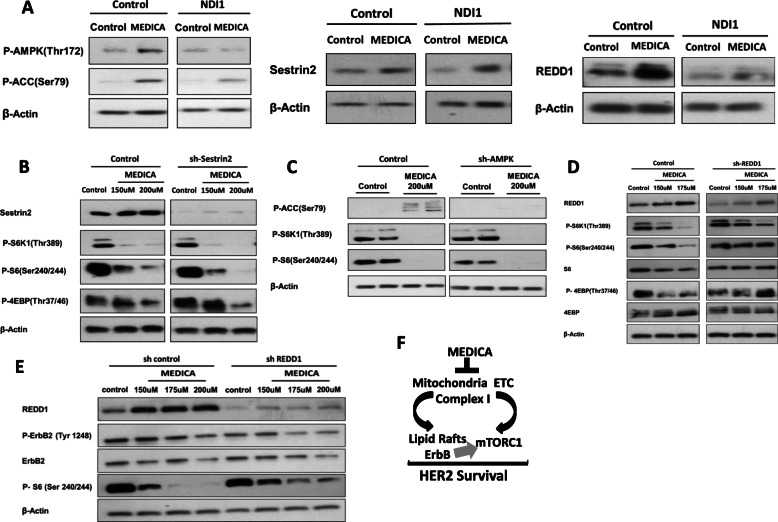
Suppression mTORC1 activity by MEDICA. **a** BT474 cells infected with empty or NDI1 virus were treated for 24 h with 150uM MEDICA as indicated. Representative blots. **b**, **c** BT474 cells infected with empty, shSestrin2, or shAMPK were treated for 28 h with MEDICA as indicated. Representative blots. **d**, **e** BT474 cells infected with control or shREDD1 were treated for 28 h with MEDICA as indicated. Representative blots. **f** Suppression of mitochondrial complex I activity by MEDICA results in suppression of mTORC1 activity and lipid raft disruption with loss of ErbB members. The double hit of MEDICA results in abrogating HER2 survival

Inhibition of mTORC1 activity due to mitochondrial stress prompted our interest in dissecting elements of the mTORC1 system that transduce inhibition of mTORC1 activity by MEDICA. mTORC1 activity may be inhibited by limiting ATP, by activating TSC1,2 and/or Raptor by AMPK, by stabilizing TSC1,2 by REDD1, and/or by modulating the activity of RAGs by Sestrin2 or lysosomal v-ATPase [43]. Indeed, MEDICA treatment resulted in induced-REDD1, phospho-AMPK(Thr172), and its ACC(Ser79) substrate, being rescued by infected NDI1 (Fig. 7a). MEDICA treatment further resulted in induced Sestrin2 (Fig. 7a). However, inhibition of mTORC1 activity by MEDICA, as verified by mTORC1 downstream phospho-S6K1(Thr389), phospho-S6(Ser240/244), and phospho-4EBP(Thr37/46) substrates, was not rescued by downregulating Sestrin2 or AMPK by respective shRNAs (Fig. 7b, c), or by constitutive RagB. Inhibition of mTORC1 activity by MEDICA was partially rescued by infected shREDD1 (Fig. 7d), indicating that TSC1,2 stabilization by MEDICA-induced REDD1 may partly account for mTORC1 inhibition by MEDICA. Of note, ErbB2 loss by MEDICA still prevails upon partially rescuing mTORC1 activity by infected shREDD1 (Fig. 7e), implying that suppression of ErbB members and inhibition of mTORC1 activity by MEDICA are independently driven by mitochondrial targeting.

## Discussion

MEDICA treatment is shown here to suppress ErbB2 breast tumors and lung metastasis in transgenic mice that express the activated ErbB2/neu oncogene under the control of the mouse MMTV long terminal repeat (LTR) promoter. Suppression of ErbB2 breast cancer by MEDICA was corroborated by suppressing ErbB2/neu xenografts in NOD/SCID mice. MEDICA effects were further verified by growth suppression of trastuzumab-sensitive and trastuzumab-resistant AU565 and BT474 human ErbB2 breast cancer cells. MEDICA inhibited the viability of ErbB2 tumor cells, colonies, and spheroids, while no resistance to MEDICA was observed in ErbB2 cells subjected to prolonged continuous culturing with MEDICA.

Suppression of ErbB2 breast tumors by MEDICA in ErbB2/neu mouse transgenes as well as in human ErbB2 breast cancer cells was accompanied by loss of plasma membrane ErbB2, together with other ErbB family members, including EGFR (ErbB1) and ErbB3. Since ErbB2 breast cancer is driven by homodimerization and/or heterodimerization of the amplified ErbB2 with other ErbB receptors, the loss of ErbB family receptors may further promote MEDICA efficacy in suppressing ErbB2 breast cancer. Loss of plasma membrane ErbB family members by MEDICA was not due to decrease in respective ErbB transcripts, but to lipid raft disruption by MEDICA, as verified by the concomitant loss of lipid raft caveolin-1 and GM1. Since stabilized lipid rafts form platforms for receptor tyrosine kinases docking and their signaling cascades [31, 32], lipid raft disruption and loss of ErbB family members by MEDICA resulted in abrogating signaling cascades downstream of ErbB receptors, including PI3K/Akt and mTORC1 as verified by its downstream targets S6K1(Thr389), S6(Ser240/244), and 4EBP(Thr37/46). Suppression of mTORC1 activity by MEDICA could apparently be ascribed to abrogation of ErbB signaling pathways leading to mTORC1. However, mTORC1 activity was inhibited by MEDICA prior to loss of ErbB receptors, while canonical mTORC1 inhibitors (rapamycin, torin) failed to disrupt ErbB members, implying that MEDICA may act as novel mTORC1 kinase inhibitor independently of abrogating the ErbB receptors and their signaling cascades (Fig. 7f).

The double hit of MEDICA in inhibiting mTORC1 activity while concomitantly abrogating ErbB signaling is noteworthy in terms of resistance to current treatment strategies. Indeed, 20% of ErbB2 breast cancer patients present primary resistance to ErbB2-targeting agents (e.g., trastuzumab, prastuzumab, lapatinib), and 70% of patients with ErbB2 metastatic cancer present acquired resistance [2]. Resistance may result from epitope masking, truncation or mutation of the ErbB2 receptor, overexpression of other ErbB family members or alternative survival receptor tyrosine kinases (RTK) (e.g., IR, IGF1R, PDGFR), and/or mutated downstream survival transduction pathways (e.g., PTEN/PI3K/Akt, MEK/MAPK). All converge onto mTORC1 and/or mTORC2, prompting treatment strategies that target mTOR. However, inhibition of mTORC1 by rapalogs may result in Akt(Thr308, Ser473) and/or MEK/MAPK phosphorylation and resistance, due to de-suppression of ErbB members and/or alternative survival RTKs and/or mTORC2 that are restrained by active mTORC1, but become activated upon mTORC1 inhibition [44–48]. Replacing rapalogs by mTOR kinase inhibitors (e.g., Torin) that inhibit both mTORC1 and mTORC2, may overcome Akt(Ser473) phosphorylation, but still result in resistance due to active phospho-Akt(Thr308) [44]. Hence, the inherent feedback that controls the ErbB2/mTOR network requires concomitant suppression of ErbB members and/or PI3K/Akt when targeting mTORC1 [48]. However, these drug combination protocols are complicated by multi-drug toxicity [49]. In contrast to mTOR canonical inhibitors, treatment of ErbB2 breast cancer by MEDICA allows for both, inhibition of mTORC1 activity while concomitantly disrupting ErbB family members and their signaling cascades (Figs. 4a–c and 6a). Hence, MEDICA may offer a novel mode of suppressing mTORC1 activity.

The bifunctional activity of MEDICA in the ErbB/mTORC1 context is partly driven by targeting mitochondria complex I. Indeed, by-passing mitochondrial complex I by infected yeast NADH dehydrogenase (NDI1) resulted in partly rescuing ErbB expression, mTORC1 activity, and cell growth. In line with that, rescue of MTORC1, ErbB2, and its signaling pathways by NDI1 infection was more evident in cells cultured in galactose medium instead of glucose, namely, under conditions that drive cells to maximize mitochondrial oxidative phosphorylation rather than relying on glycolytic ATP production [38, 50]. The mitochondrial metabolic stress induced by MEDICA may result in suppressing mTORC1 activity by promoting lysosomal/TSC1,2 association [35]. Indeed, suppression of mTORC1 activity by MEDICA was rescued by abrogating REDD1, implying that TSC1,2 stability is required for MEDICA activity in suppressing mTORC1. Lipid raft disruption with loss of caveolin-1 and ErbB receptors has recently been reported to be mediated by NDRG1 overexpression [51, 52], that could in principle be induced by mitochondrial metabolic stress [53]. However, no increase in NDRG1 expression was evident in ErbB2 breast cancer cells cultured in the presence of MEDICA. The punctuated GM1 appearance of MEDICA-treated cells may indicate clustering of lipid rafts due to oxidative stress [33].

Retrospective clinical studies have indicated improved disease-free and overall survival of diabetic ErbB2 luminal B breast cancer patients treated with metformin [10–13]. The efficacy of add-on metformin in the treatment of ErbB2 breast cancer in non-diabetic patients still remains to be prospectively verified. Of note, add-on metformin to neo-adjuvant treatment of ErbB2 breast cancer in non-diabetic patients has recently been reported to fail in improving pathologic complete response [54], indicating perhaps metformin limitation in the non-diabetic breast cancer context. In light of the role of hyperinsulinemia in breast cancer growth and migration [55], the robust insulin-sensitizing anti-diabetic activity of MEDICA [17, 18, 20, 21] may translate to improved treatment of ErbB2 breast cancer in both diabetic and non-diabetic patients.

## Conclusions

In spite of the advances accomplished in treating ErbB2 breast cancer, the ErbB2 cancer challenge still remains an unmet need. Also, mTORC1 is activated in up to 80% of human cancers [56], while current mTOR inhibitors are of limited clinical efficacy due to resistance invoked by de-suppression of mTOR feedback networks and due to worrisome side effects. If proved in future clinical studies, the bifunctional activity of MEDICA in abrogating ErbB receptors and inhibiting mTORC1 activity (Fig. 7f) may offer a novel treatment mode for mTORC1-driven cancers in general and ErbB2 breast cancer in particular.

## Supplementary information

**Additional file 1:.** Supplemental figures

## Data Availability

All data generated or analyzed during this study are included in this published article.

## Abbreviations

AMPK: AMP-activated protein kinase
FCS: Fetal calf serum
LCFA: long-chain fatty acids
MMTV: Mouse mammary tumor virus
NDI1: Yeast NADH dehydrogenase
PyMT: Polyoma middle T antigen
RTK: Receptor tyrosine kinases
T2D: Type 2 diabetes
