# Supplementary material for: Treatment of ErbB2 breast cancer by mitochondrial targeting
Source: Cancer Metab. 2020 Jul 14;8:17. doi: 10.1186/s40170-020-00223-8 (PMC7362624; doi:10.1186/s40170-020-00223-8)

LEGENDS TO SUPPLEMENTAL FIGURES

Figure 1. Growth inhibition of ErbB2 breast cancer cells by mitochondrial complex I inhibitors.
AU565 (**A**) and BT474 (**B**) cells were grown in DMEM medium and treated for 96h as indicated. Cell growth (O.D 630) Mean ± SD.

Figure 2. Growth inhibition of A549 cancer cells by mitochondrial complex I inhibitors.
A549 cells were grown in DMEM medium and treated for 96h as indicated. Cell growth (O.D 630) Mean ± SD.

Supp Figure 1

A

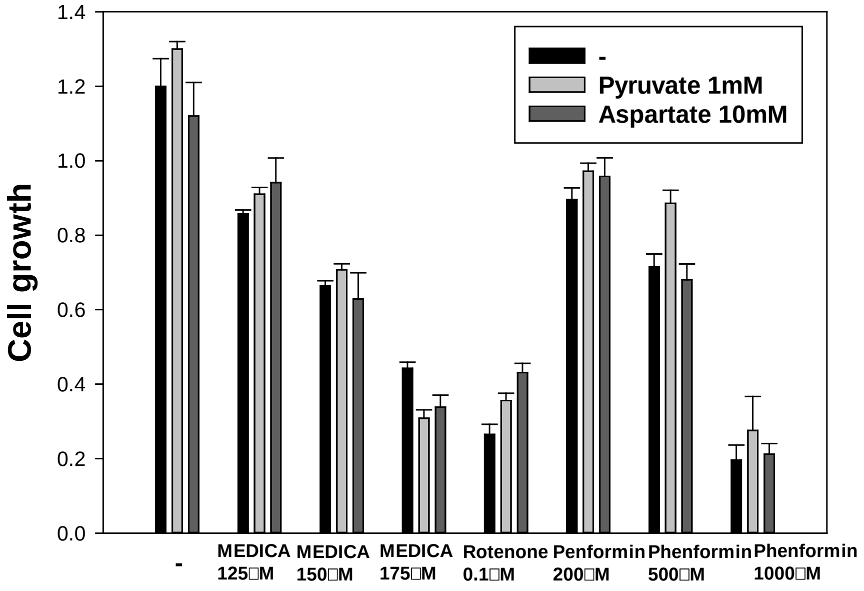

B

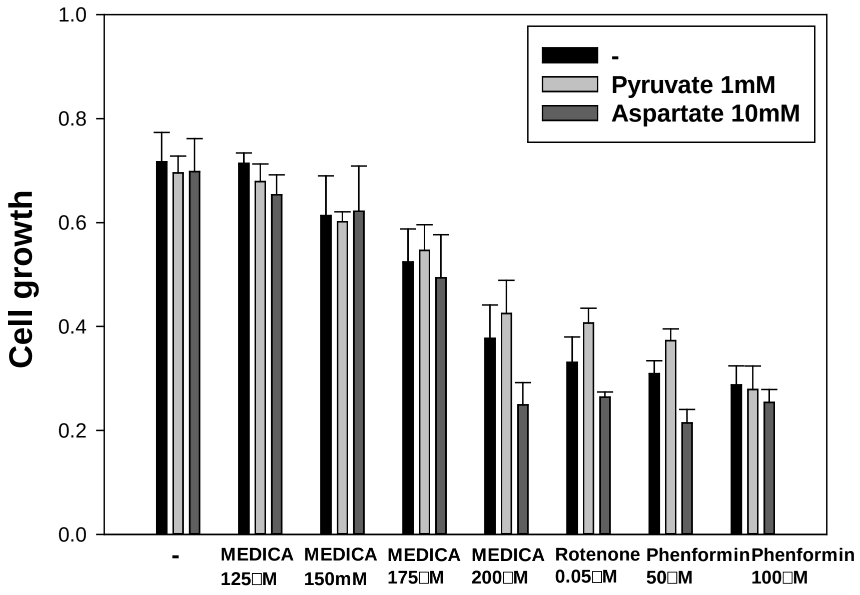

Supp Figure 2

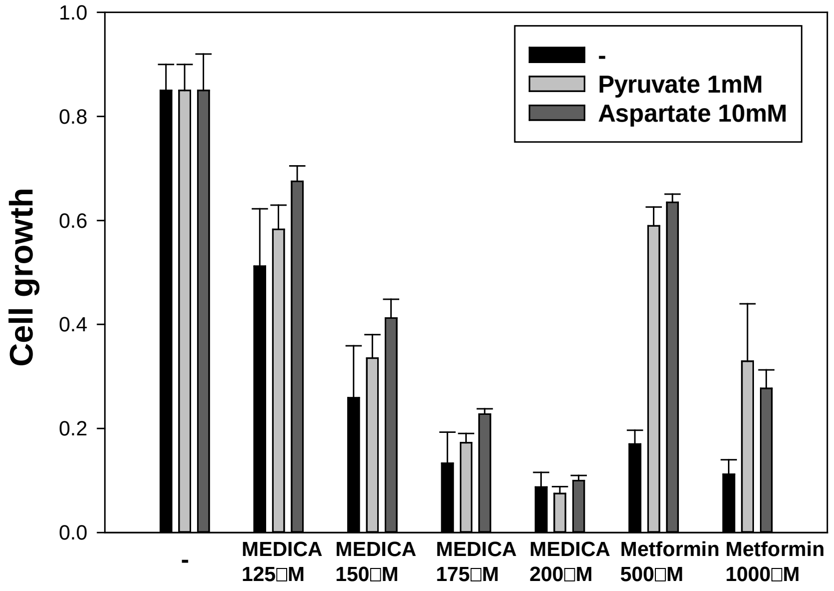

Supplement: Supplementary file 1 — Additional file 1:. Supplemental figures [file 40170_2020_223_MOESM1_ESM.pdf]
